# Difference in Acquired Radioresistance Induction Between Repeated Photon and Particle Irradiation

**DOI:** 10.3389/fonc.2019.01213

**Published:** 2019-11-12

**Authors:** Katsutoshi Sato, Takashi Shimokawa, Takashi Imai

**Affiliations:** ^1^Division of Hematology and Medical Oncology, Icahn School of Medicine at Mount Sinai, The Tisch Cancer Institute, New York, NY, United States; ^2^Department of Accelerator and Medical Physics, National Institute of Radiological Sciences, National Institutes for Quantum and Radiological Sciences and Technology, Chiba, Japan; ^3^Medical Databank, Department of Radiation Medicine, QST Hospital, National Institutes for Quantum and Radiological Science and Technology, Chiba, Japan

**Keywords:** cancer, radioresistance, acquisition, X-ray radiation, carbon-ion radiation, repeated irradiation, DNA repair, aggressiveness

## Abstract

In recent years, advanced radiation therapy techniques, including stereotactic body radiotherapy and carbon–ion radiotherapy, have progressed to such an extent that certain types of cancer can be treated with radiotherapy alone. The therapeutic outcomes are particularly promising for early stage lung cancer, with results matching those of surgical resection. Nevertheless, patients may still experience local tumor recurrence, which might be exacerbated by the acquisition of radioresistance after primary radiotherapy. Notwithstanding the risk of tumors acquiring radioresistance, secondary radiotherapy is increasingly used to treat recurrent tumors. In this context, it appears essential to comprehend the radiobiological effects of repeated photon and particle irradiation and their underlying cellular and molecular mechanisms in order to achieve the most favorable therapeutic outcome. However, to date, the mechanisms of acquisition of radioresistance in cancer cells have mainly been studied after repeated *in vitro* X-ray irradiation. By contrast, other critical aspects of radioresistance remain mostly unexplored, including the response to carbon-ion irradiation of X-ray radioresistant cancer cells, the mechanisms of acquisition of carbon-ion resistance, and the consequences of repeated *in vivo* X-ray or carbon-ion irradiation. In this review, we discuss the underlying mechanisms of acquisition of X-ray and carbon-ion resistance in cancer cells, as well as the phenotypic differences between X-ray and carbon-ion-resistant cancer cells, the biological implications of repeated *in vivo* X-ray or carbon-ion irradiation, and the main open questions in the field.

## Introduction

The previous decade has seen significant developments in the techniques used in radiotherapy, and advanced radiotherapy has become increasingly adopted. Among advanced radiotherapy techniques, stereotactic body radiation therapy (SBRT) relies on a small irradiation field to precisely deliver high doses of radiation, typically above 10 Gy per fraction, to local tumors. SBRT has been applied to the treatment of various cancers, including lung (1), liver (2), and prostate cancer (3). The therapeutic outcomes of SBRT are particularly promising for early stage lung cancers, with local control rates exceeding 80% (1, 4) and clinical outcomes comparable to those of surgical resection (5, 6).

In addition to SBRT, particle-beam therapy, such as carbon-ion (C-ion) radiotherapy (CIRT), has demonstrated excellent therapeutic outcomes in various types of cancer (7). CIRT has both physical and biological advantages compared with X-ray therapy. With CIRT, tumors are irradiated with C-ions of relativistic energy, which means that C-ions penetrate the body with lower ionization that significantly increases toward the end of the beam path. This radiophysical feature is commonly referred to as the “Bragg-peak” and contributes to increasing the radiation dose delivered to the tumor while minimizing the radiation dose delivered to the skin and surrounding healthy tissues. Furthermore, CIRT has a relative biological effectiveness, which is defined as the ratio of a dose of radiation to the dose of X-ray radiation producing the same biological effects that is >2. Another important feature of CIRT is its effectiveness against conventional X-ray radiotherapy resistant cancers, including melanoma (8) and bone and soft-tissue sarcoma (9–11). Furthermore, CIRT reportedly works as an alternative ablative treatment for early stage lung cancer, in particular for elderly and inoperable patients (12).

Nevertheless, recent studies show that local recurrence can still occur after advanced radiotherapy. For example, an incidence of local recurrence ranging from 4.9 to 19% in patients who received SBRT for lung cancer treatment was reportedly dependent on treatment regimen, tumor stage, and follow-up periods (13–18). Furthermore, 23.3% of patients who received CIRT for the treatment of stage I non-small cell lung cancer also exhibited local recurrence (19).

In cases of tumor recurrence after primary radiotherapy, patients can rarely be treated again with the same radiation regimen, because the tumor might acquire radioresistance, and it is possible that healthy surrounding tissues will not tolerate additional irradiation. Nevertheless, recent studies report that SBRT and CIRT can be used for re-irradiation of recurrent tumors, taking into account both the dose tolerance of healthy tissues and location of the recurrent tumor (20–23). However, several issues related to re-irradiation with SBRT or CIRT still need to be considered. First, only a few studies have reported the clinical outcomes of repeated irradiation, and second, the characteristics of recurrent tumor after primary radiotherapy are largely unknown. In this review, we focus on the biological aspects of acquired X-ray and C-ion resistance in cancer cells and discuss the differences between the consequences of *in vitro* and *in vivo* repeated irradiation, the possible mechanisms of acquired resistance in cancer cells, and issues that must be addressed in this research field.

## Acquisition of Photon Radioresistance *in vitro*

Radioresistance acquisition in cancer cells and its underlying mechanisms have been mainly studied using radioresistant cell lines established through repeated *in vitro* photon (e.g., X-ray or γ-ray) irradiation. Because conventional radiotherapy usually relies on a total dose of ~60 Gy applied in 2-Gy fractions, many studies adopted similar radiation regimens in order to establish radioresistant cell lines (Table 1) (24–41). Importantly, most of these studies showed that the survival of repeatedly irradiated cells was significantly higher than that of the parental cells, which indicated that *in vitro*, various cell lines could acquire radioresistance following multiple rounds of X-ray irradiation.

**Table 1:** Repeated photon irradiation regimen for the establishment of radioresistant cancer cells.

**Author (year)**	**Single dose (Gy)**	**Total dose (Gy)**	**Regimen**	**Parental cell line**	**Main findings**	**References**
Kuwahara et al. (2009, 2011)	0.5	>1,600	2 Gy of α-ray, 0.5 Gy/12 h	HepG2	•Promoted DNA repair •Decreased autophagic cell death	(24, 25)
Lee et al. (2010)	2	80	Over 5 months	H460	•Decreased reactive oxygen species production •TP53I3 downregulation	(26)
Lin et al. (2010)	2	60	^(*1)^	OECM1 KB SAS	•GP96 upregulation	(27)
Luo et al. (2017)	2	30	^(*1)^	TE-1 Eca-109	•Concomitant increase in CDDP resistance	(28)
Lynam-Lennon et al. (2010)	2	50	^(*1)^	OE33	•Promoted DNA repair	(29)
Mitsuhashi et al. (1996)	6.37	63.7	^(*1)^	NMT-1	•^(*2)^	(30)
Pearce et al. (2001)	2–4	40–60	Weekly	MDA-MB-231	•^(*2)^	(31)
Post et al. (2018)	1–4	64	4 Gy/2 weeks, 4 times/week	MCF7	•^(*2)^	(32)
Russell et al. (1995)	2	50	Every 5–7 days	IMR32	•Promoted DNA repair	(33)
Sato et al. (2014, 2017)	10, 5 ^(*3)^	60, 30 ^(*3)^	Every 2 weeks	NR-S1	•Promoted DNA repair •Resistance to C-ion radiation •Increased phosphorylation of mTOR	(34, 35)
Shimura et al. (2010, 2014, 2017)	0.5	31–62	Every 12–24 h	Hela HepG2	•Activation of the DNA-PK-Akt-Cyclin D1 pathway	(36–38)
Shintani et al. (2011)	2	60	Over 6 months	A549	•Induction of epithelial to mesenchymal transition	(39)
You et al. (2014)	2	80	40 times over 5 months	A549 H157 H358	•Increased phosphorylation of JAK2 and STAT3 •Increased levels of Bcl2 and Bcl2-XL	(40)
Zhou et al. (2010)	6.37	76.44	12 times over 6 months	Hep2	•Increased fraction of cells in the G0 phase •Increased telomerase activity	(41)

(*1) *There is no description about treatment regimen*.

(*2) *There is no significant finding other than radioresistance acquisition*.

(*3) *5 Gy and 30 Gy of single and total dose is that of C-ion irradiation*.

### Cellular Processes Involved in the Acquisition of Radioresistance Following Repeated Photon Irradiation

The mechanisms of acquisition of radioresistance in cancer cells have been associated with a variety of biological processes (Table 1). However, in many cases, the acquisition of radioresistance can be reasonably explained by the induction of epithelial-to-mesenchymal transition (EMT), which is defined as phenotypic and molecular alterations that result in the loss of epithelial-cell characteristics and the gain of mesenchymal-cell characteristics. As cancer cells undergo EMT, epithelial markers, such as E-cadherin, ZO-1, and cytokeratin, are downregulated, whereas mesenchymal markers, such as N-cadherin, vimentin, snail, and twist, are upregulated, and in some cases, morphological changes lead to the appearance of spindle-shaped cells (42). The most prominent characteristics acquired by cancer cells after EMT are migratory and invasive properties, conferring them a significant metastatic potential, and resistance to chemotherapeutic drugs and ionizing radiation. Indeed, several studies report that EMT in cancer cells, which was defined by reduced E-cadherin protein levels, increased N-cadherin protein levels, and enhanced migration potential, could be induced by single X-ray irradiation (43, 44). Furthermore, Shintani et al. (39) showed that repeated X-ray irradiation of A549 cells (2 Gy weekly for >6 months) induced significant radioresistance and typical EMT (i.e., decreased E-cadherin and increased N-cadherin mRNA and protein levels). Collectively, these data support the notion that EMT induction following X-ray irradiation is a contributing factor in the acquisition of radioresistance.

Another factor involved in the acquisition of radioresistance following repeated X-ray irradiation is an enrichment in cancer stem cells (CSCs), which are known to exhibit higher DNA-repair potential (45) and resistance to reactive oxygen species-induced cytotoxicity (46, 47). CSCs are also often found to be in the G0 phase (48), a quiescent state outside the normal cell cycle and associated with reduced cell proliferation. All of these characteristics are recognized for their role in cellular radioresistance. Additionally, CSCs are important in radioresistance acquisition because of their impact on the heterogeneity of the cell population within a tumor. Indeed, in the hierarchy model, CSCs produce a more differentiated non-CSC progeny exhibiting significant cell-proliferation potential but lacking stem cell properties. Notwithstanding their reduced proliferation rate as compared with their non-CSC progeny, CSCs can self-renew, and maintaining their stemness. Notably, the non-CSC population displays higher radiosensitivity than the CSC population. Consequently, radiation treatment can increase the relative abundance of CSCs in the tumor, which promotes asymmetric cell proliferation and, therefore, an enrichment in CSCs.

Lagadec et al. (49) reported enrichment in CD44^high^/CD24^low^ breast CSCs following repeated X-ray irradiation of human breast cancer MCF7 and T47D cells, with irradiated cells displaying increased sphere-formation potential. Furthermore, they found that repeated irradiations led CSCs in the G0 phase to reenter the cell cycle, thereby promoting their proliferation, whereas the non-CSC population underwent apoptosis according to the increased fraction of cells in the sub-G1 phase (49). Additionally, Ghisolfi et al. (50) showed that single X-ray irradiation of cancer cells with a dose of 2 Gy to 10 Gy increased the expression of the pluripotency markers *OCT3/4* and *SOX2* and promoted the enrichment of a CSC subpopulation. Moreover, Mani et al. (51) established a link between EMT and CSCs by demonstrating that TGF-β-induced EMT generated a subpopulation with CSC properties, including characteristic CSC markers, such as CD44^high^/CD24^low^ and elevated sphere- and mammosphere-formation potential.

To the best of our knowledge, a definitive mechanism responsible for the induction of CSCs remains unclear; however, DNA damage or chromosomal aberration can enhance CSC induction along with increased oncogene activity. Liang et al. (52) showed that DNA damage from UV irradiation and the chromosomal aberrations induced by *Mad2* overexpression also increased by *Myc* and *SOX2* expression in human nasopharyngeal carcinoma CNE cell lines and promoted cell dye-exclusion, colony formation, and sphere-formation capacities. These data suggest that the accumulation of DNA damage by repeated X-ray irradiation induces not only EMT but also enrichment of CSCs with increasing oncogenic activity, whereas secondary induction of a CSC subpopulation by EMT (known as cancer plasticity) further contributes to the development of radioresistance.

### Molecular Processes Involved in the Acquisition of Radioresistance Following Repeated Photon Irradiation

**Figure 1:**
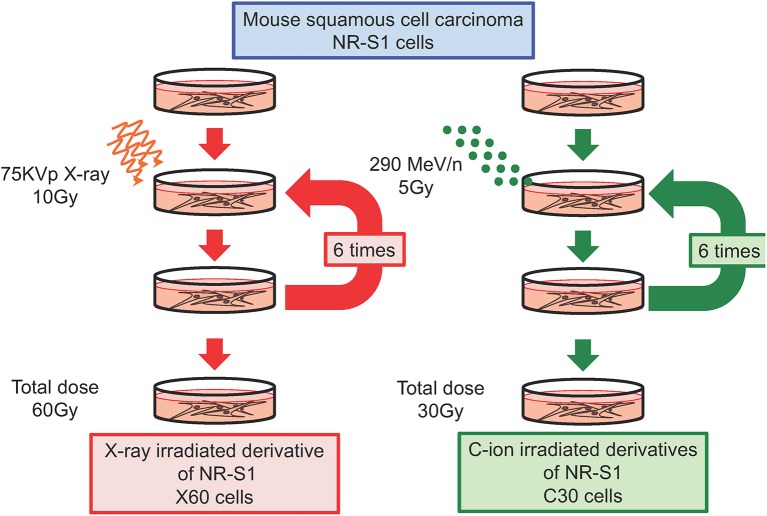
Diagram describing the establishment of radioresistant cancer cells through repeated X-ray or C-ion irradiation. Mouse squamous cell carcinoma NR-S1 cells were irradiated six times at 2-week intervals with 10 Gy of X-ray radiation (left) or 5 Gy of C-ion radiation (left). The radioresistant derivative cell lines exposed to total doses of 60 Gy of X-ray radiation and 30 Gy of C-ion radiation were denoted as X60 and C30 cells, respectively (34, 35).

We and others have independently reported that repeated X-ray irradiation can result in enhanced DNA-repair capacity (24, 29, 33, 34). In our study, the mouse squamous cell carcinoma NR-S1 cell line was irradiated with a total dose of 60 Gy of X-ray radiation applied in 10-Gy fractions in order to establish the X60 radioresistant cancer cell line (Figure 1). Notably, the D10 value (i.e., the radiation dose required to decrease the survival to 10% of the non-irradiated condition) and cell survival after 10 Gy of X-ray radiation were 1.6- and 3.8-fold higher, respectively, for X60 cells than for parental NR-S1 cells (34). Furthermore, 24 h after exposure to 10 Gy X-ray radiation, the number of S139 phosphorylated-H2AX (γ-H2AX) foci, a marker of DNA double-strand breaks (DSBs), was 2.5-fold lower in X60 cells than in NR-S1 cells, indicating that DSBs were repaired more efficiently in X60 cells than in NR-S1 cells (34). Indeed, the collected results of numerous studies (Table 1) further demonstrate that enhanced DNA-repair capacity is a common feature of radioresistant cancer cells arising from repeated X-ray irradiation.

As part of the investigation of the molecular mechanisms underlying the acquisition of radioresistance, several groups have highlighted a relationship between DNA repair and pro-survival signaling pathways, such as the Akt and mechanistic target of rapamycin (mTOR) pathways. Shimura et al. (36) suggested a potential molecular mechanism for the acquisition of radioresistance induced by repeated X-ray irradiation, showing that *cyclin D1* expression and Akt phosphorylation levels were increased in X-ray-resistant derivatives of HeLa and HepG2 cells established following repeated irradiation. These radioresistant cancer cell lines also displayed constitutively elevated levels of DSBs, as measured by H2AX and ataxia telangiectasia mutated (ATM) phosphorylation, relative to those in parental cell lines. Strikingly, downregulation of cyclin D1 in radioresistant HeLa and HepG2 derivatives decreased H2AX-, ATM-, and Akt-phosphorylation levels, as well as cell survival, after further X-ray irradiation. Therefore, they proposed that repeated X-ray irradiation triggered *cyclin D1* overexpression and forced cell cycle progression, which in turn caused further DNA damage and led to the activation of both Akt signaling and DNA-dependent protein kinase activity, a central component in the non-homologous end joining DSB-repair pathway. Eventually, these signals promoted further *cyclin D1* overexpression as part of a positive-feedback loop that likely resulted in the acquisition of radioresistance (36, 38, 53, 54).

In addition to Akt signaling, mTOR signaling has been associated with the acquisition of X-ray resistance in cancer cells. Chang et al. (55) established radioresistant derivatives of PC-3, DU145, and LNCaP cells following repeated X-ray irradiation with a dose of 2 Gy/day for 5 consecutive days (55) and showed that these radioresistant cancer cell lines exhibited both mesenchymal and CSC phenotypic traits. Interestingly, they found that radioresistant cells treated with BEZ235, a specific inhibitor of the phosphoinositide 3 (PI3) and mTOR kinases, displayed decreased expression of mesenchymal (N-cadherin, vimentin, and snail) and CSC (OCT3/4, SOX2, and CD44) markers, increased expression of the epithelial marker E-cadherin, and reduced cell survival. They further showed that BEZ235, the PI3-kinase inhibitor BKM120, and the mTOR-kinase inhibitor rapamycin, suppressed the expression of DNA-repair proteins induced by X-ray irradiation, including Ku80, BCRA1, and BRCA2 (56). Moreover, we independently demonstrated that mTOR signaling was enhanced in X60 radioresistant cancer cells as compared with parental NR-S1 cells, whereas rapamycin treatment decreased their radioresistant phenotype (35). Importantly, rapamycin also suppresses both non-homologous end joining and homologous recombination (HR) DSB-repair pathways (57). Collectively, these results indicate that activation of the pro-survival Akt and mTOR signaling pathways can eventually increase the DNA-repair capacity of repeatedly irradiated cancer cells and thereby promote the acquisition of radioresistance.

## Acquisition of C-ion Radioresistance *in vitro*

### Acquisition of C-Ion Resistance Following Repeated X-Ray Irradiation

In clinical practice, CIRT is an effective treatment for locally recurrent tumors after primary radiotherapy, likely because cell killing by C-ion radiation is independent of various cellular or tumor characteristics, including p53 status (58), cell cycle phase (59, 60), and hypoxia (61–63). Although these findings suggest that C-ion radiation should be effective against radioresistant cancer cells arising from repeated X-ray irradiation, no available experimental data supported this hypothesis. Therefore, in a recent study, we determined whether C-ion radiation could efficiently kill X60 radioresistant cancer cells. Contrary to our expectations, we found that compared with parental NR-S1 cells, X60 cells exhibited significant levels of resistance against C-ion radiation (34). Furthermore, 24 h after C-ion irradiation, the number of γ-H2AX foci was 2.5-fold lower in X60 cells than in NR-S1 cells. These observations indicated that repeated X-ray irradiation of cancer cells with a relatively high dose of 10 Gy per fraction could induce not only X-ray resistance but also C-ion resistance (34). We believe that further investigations using such radioresistant cells will likely lead to the discovery of novel mechanisms contributing to C-ion resistance in cancer cells.

To gain further insight into the underlying mechanisms of radioresistance in X60 cells, in a recent study, we compared several biological and morphological traits of X60 cells and parental NR-S1 cells, including cell shape and size, number of heterochromatin domains in the nucleus, and DNA content (34). Additionally, we analyzed the correlation between these factors and X-ray or C-ion resistance. Interestingly, we found that the number of heterochromatin domains was strongly correlated with both X-ray and C-ion resistance, which suggested that heterochromatin components or the dynamics of heterochromatin were also involved in the acquisition of radioresistance.

**Figure 2:**
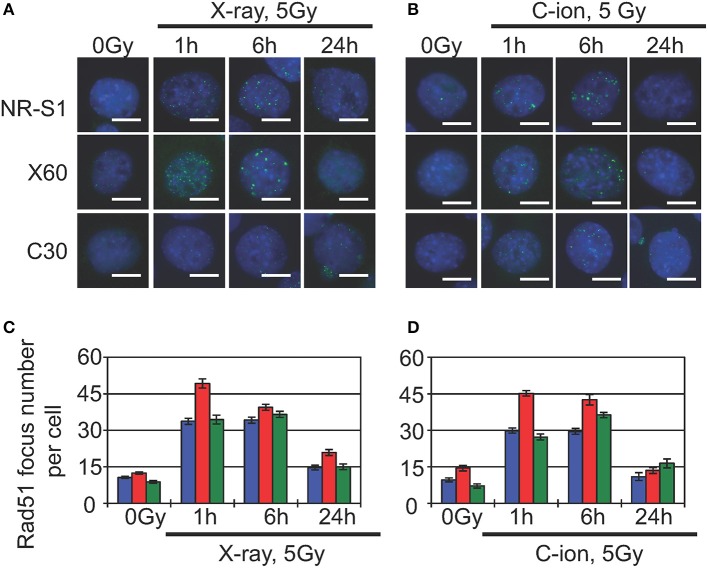
Analysis of Rad51-foci formation in parental NR-S1 cells and radioresistant X60 and C30 cells. **(A,B)** Representative immunofluorescence images of NR-S1, X60, and C30 cells irradiated with 10 Gy of X-ray radiation **(A)** or 5 Gy of C-ion radiation **(B)**, as previously described (51, 56). At the indicated time after irradiation, cells were fixed and labeled with anti-Rad51 antibodies (green) using standard procedures (34, 35). Nuclei were counterstained by Hoechest33342 (blue). Scale bars, 10 μm. **(C,D)** Histograms showing the average number of Rad51 foci per cell following X-ray **(C)** and C-ion **(D)** irradiation of NR-S1 (blue), X60 (red), and C30 (green) cells. Data represent the mean ± standard deviation.

Indeed, previous studies show that heterochromatin proteins, such as HP1α (64) and CAF1 (65), are directly involved in DNA repair, and recent studies report that DNA damage in heterochromatin domains is mainly repaired by the HR machinery (66, 67). The damaged heterochromatin first move to the periphery of the heterochromatin domain to prevent abnormal recombination or deleterious expansion at satellite or repetitive DNA sequences, after which Rad51, the core component of the HR machinery, accumulates at DNA-damage sites located at the periphery of the heterochromatin domain (66, 67). Although these studies were conducted in *Drosophila* and yeast cells, Jakob et al. (68) observed similar dynamics of damaged heterochromatin and DNA-repair following heavy ion-beam irradiation of mammalian cells, finding that immediately after irradiation, DSBs were formed in the heterochromatin along the uranium ion-beam track. Within 30 min, the DSB sites relocated to the periphery of the heterochromatin domain, and replication protein A, a marker of DNA-end resection during HR repair, accumulated at these DSB sites (68). In agreement with these findings, our preliminary results showed that 1 h after X-ray or C-ion irradiation, X60 cells displayed an increased number of Rad51 foci as compared with NR-S1 cells (Figure 2). Because Rad51 is a central factor in the HR machinery, Rad51 foci are commonly considered to represent sites of ongoing DSB repair by HR.

Furthermore, several studies report that HR repair can remove complex DNA lesions, including clustered DNA damage (69) and DNA-protein crosslinks (70, 71). Given that X60 cells exhibit increased numbers of heterochromatin domains and radiation-induced Rad51 foci as compared with NR-S1 cells (Figure 2), it appears conceivable that enhanced HR-repair capacity and heterochromatin dynamics contribute to X-ray and C-ion resistance in X60 cells. Whether such a mechanism of acquisition of radioresistance could be specific to X60 cells or shared by other radioresistant cancer cell lines remains to be explored. Nevertheless, future studies focusing on further our understanding of the underlying mechanisms of DSB repair in heterochromatin domains could, therefore, represent a significant breakthrough toward elucidating the mechanisms of acquisition of radioresistance in cancer cells and identifying novel therapeutic strategies for the treatment of radioresistant tumors.

### Acquisition of C-Ion Resistance Following Repeated C-Ion Irradiation

To date, only a limited number of studies have investigated the acquisition of C-ion resistance, and it remains unclear whether repeated C-ion irradiation can lead to radioresistance in cancer cells. To address this question, in a recent study, we irradiated NR-S1 cells with a total dose of 30 Gy of C-ion radiation applied in 5-Gy fractions in order to establish a C30 radioresistant cancer cell line (Figure 1). This C-ion irradiation regimen is biologically equivalent to the X-ray irradiation regimen used to establish the X60 cell line, because NR-S1 cells exhibit comparable survival following exposure to 5 Gy and 10 Gy of C-ion and X-ray radiation, respectively. Interestingly, when we assessed the X-ray and C-ion radiation sensitivity of C30 cells using a colony formation assay, we found that C30 cells displayed moderate resistance to C-ion radiation but not to X-ray radiation (35).

Although it remains unclear why repeated X-ray irradiation but not repeated C-ion irradiation conferred significant C-ion-resistance to NR-S1 derivative-cells, we believe that enrichment in CSCs might be a contributing factor. Indeed, it is widely recognized that CSCs are more resistant to X-ray radiation and anticancer drugs than more differentiated cancer cells. Furthermore, as noted, several studies report that repeated X-ray irradiation can increase the CSC fraction within a cancer cell population (49, 50, 72–74). Conversely, Cui et al. (75) showed that C-ion radiation could efficiently kill CSCs both *in vitro* and *in vivo*. Therefore, it is conceivable that repeated X-ray irradiation but not repeated C-ion irradiation could contribute to enriching a radioresistant subpopulation with CSC-like characteristics. However, there is one element in contradiction to this hypothesis. Because most CSCs are in a G0 quiescent state (45), the non-CSC subpopulation might proliferate faster; therefore, if the primary factor in the acquisition of X-ray and C-ion resistance is enrichment in CSCs, the level of radioresistance of a growing X60 cell population, for example, could gradually decrease over time, which was not observed (34, 35).

Collectively, these findings indicate that CSC enrichment and other mechanisms, such as genetic alterations and mutations (76), might jointly contribute to the acquisition of both X-ray and C-ion resistance in cancer cells. Although further investigations are required to elucidate these mechanisms, the current body of evidence suggests that C-ion irradiation does not induce radioresistance and can be used for the treatment of locally recurrent tumors arising after primary CIRT.

## Effects of *in vivo* Repeated Photon or C-ion Irradiation

Numerous studies are focused on translating results obtained with *in vitro* models of radioresistant cancer cells into clinical practice. Therefore, it appears essential to determine whether phenotypic changes resulting from repeated photon or particle irradiation also occur *in vivo*. However, to the best of our knowledge, the effects of *in vivo* repeated photon or particle irradiation on the acquisition of radioresistance in cancer cells has not been reported.

**Figure 3:**
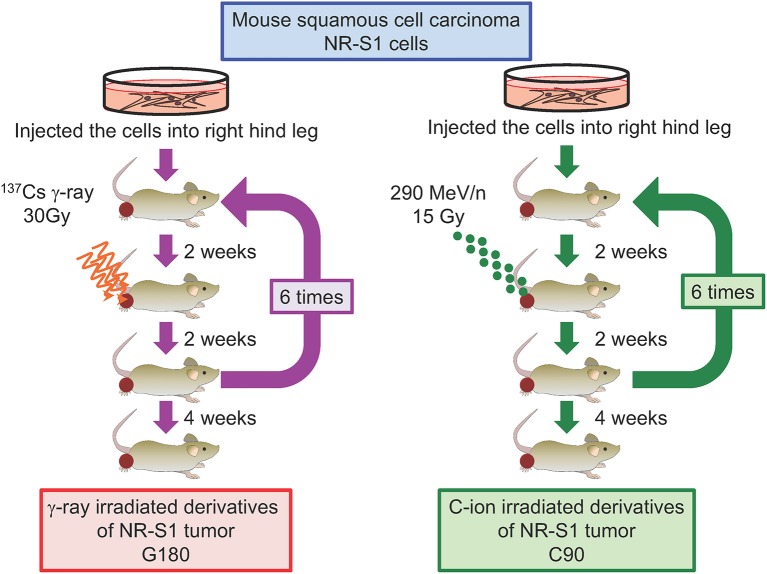
Diagram describing the establishment of regrown tumor models through repeated γ-ray or C-ion irradiation. Mouse squamous cell carcinoma NR-S1 cells were injected into the right hind leg of healthy C3H/He mice. Upon reaching ~10 mm in diameter, tumors derived from NR-S1 cells were irradiated with 30 Gy of γ-ray radiation or 15 Gy of C-ion radiation and allowed to regrow for 2 weeks before transplant into the right hind leg of healthy mice. The irradiation/regrow/transplant procedure was performed six times in total, resulting in regrown tumors exposed to total doses of 180 Gy of γ-ray radiation and 90 Gy of C-ion radiation, respectively. The tumors were finally harvested for analysis 4 weeks after the final irradiation (77).

To address this question and examine whether the characteristics of repeatedly irradiated tumors differ from those of parental tumors, we recently established *in vivo* models of regrown irradiated tumors (77). To this end, NR-S1-derived tumors engrafted into C3H/He mice were irradiated with single doses of γ-ray (30 Gy) or C-ion (15 Gy) radiation, which have comparable effects on tumor growth. Two weeks after irradiation, we harvested the irradiated tumors and transplanted them into healthy mice, and 2 weeks later, the regrown tumors were irradiated again, with this irradiation/regrowth/transplant process repeated six times in total. The resulting repeatedly irradiated tumors were exposed to total doses of 180 Gy of γ-ray radiation and 90 Gy of C-ion radiation and denoted as G180 and C90 *in vivo* regrown tumor models, respectively (Figure 3). We then examined differences in tumor-growth potential, spontaneous metastasis from the primary site to the lung surface, tumor-grafted mouse survival, and radiosensitivity between non-irradiated NR-S1-derived tumors and G180 and C90 tumors.

Notably, G180 tumors displayed drastically increased tumor-growth rates and metastatic potential compared with those of non-irradiated tumors, and mice grafted with G180 tumors displayed significantly shorter survival than those grafted with non-irradiated tumors. By contrast, the characteristics of the C90 tumors remained comparable to those of non-irradiated tumors. Importantly, X-ray and C-ion irradiation of G180 and C90 tumors did not affect the relative tumor-growth rates, spontaneous lung metastasis, and survival of tumor-grafted mice as compared with non-irradiated tumors. Furthermore, colony formation assays performed using cells isolated from non-irradiated, G180, and C90 tumors showed that they all added similar sensitivity to X-ray and C-ion radiation (77). Moreover, compared with non-irradiated and C90 tumors, G180 tumors harbored numerous microvessels and expressed genes associated with angiogenesis and metastasis, including *VEGFA, HIF1A, FN1, MMP2, MMP9, PAI1*, and *PLAU*. Together, these data indicated that, contrary to repeated *in vitro* irradiation, repeated *in vivo* γ-ray and C-ion irradiation did not lead to the acquisition of radioresistance in regrown tumors. However, repeated photon irradiation but not particle irradiation appeared to enhance tumor growth and metastasis, resulting in an increased aggressiveness of regrown tumors.

These findings could suggest that repeated irradiation affects the tumor microenvironment rather than the tumor itself or its CSC subpopulation. Although our investigations could not determine whether repeated photon irradiation enriched the CSC subpopulation in regrown tumors, our data showed that G180 tumor cells in suspension culture had a significantly higher sphere-formation potential than their non-irradiated and C90 counterparts (77). Because CSCs are characterized by significant resistance to cytotoxic agents, including radiation and anticancer drugs, we believe that G180 tumors are likely enriched in tumor-initiating cells (TICs) that differ from a typical CSC subpopulation. To date, such phenomena have not been reported, and further studies will be required to determine which cells among cancer and stromal cells are mostly affected by repeated *in vivo* irradiation and what mechanisms lead to increased aggressiveness in repeatedly irradiated tumors.

Crucially, these findings also demonstrated that repeated C-ion irradiation was far less prone to induce acquisition of radioresistance and enhance tumor aggressiveness, as assessed by tumor growth, metastatic potential, and prognosis of tumor-grafted mice. Although it remains necessary to ascertain why C-ion radiation effectively suppressed tumor aggressiveness and TIC or CSC subpopulations, we believe that the accumulated evidence supports CIRT as a promising treatment for local recurrent tumors.

## Relationship Between the 4Rs of Radiotherapy and Radioresistance Acquisition

Tumor shrinkage by fractionated radiotherapy has been explained by the “4Rs” of radiotherapy, where each “R” represents “Repair,” “Repopulation,” “Redistribution,” and “Reoxygenation” (78). “Repair” denotes the difference in cell survival of tumor cells and normal tissue between single or fractionated irradiation at the same radiation dose (79) and is basically measured by colony formation assay, followed by calculation of α and β values using a linear-quadratic model to quantify the radiosensitivity of each cell (80). “Repopulation” describes regeneration of normal tissue, such as skin and mucosal tissue (81). This concept relies on experimental results showing that the recovery of skin and mucosal tissue occurs faster than regrowth of gross tumor mass, and that each fractionation regime of radiotherapy can be determined based on these differences. Because α and β values and difference in recovery between tumor and normal tissue are used for treatment planning of radiotherapy, they are recognized as important therapeutic components. “Redistribution” indicates synchronization of the cell cycle in cells exposed to radiation. The tumor harbors multiple cell types exhibiting various cell cycle phases (82). Upon treatment of the tumor with radiotherapy, the relatively radiosensitive cell fractions, such as those in the G2/M phase, will die first, whereas the relatively radioresistant cell fractions, such as those in the G1 and S phases, will survive. However, cells surviving the first round of irradiation will enter a radiosensitive phase of the cell cycle during subsequent rounds and eventually will subsequently be efficiently killed. “Reoxygenation” describes changes in well-oxygenated areas of an irradiated tumor (83). Partial oxygen pressure in the peripheral tumor is higher than that in the center, because nutrition and oxygen at the periphery is well-supplied by tumor blood vessels (78). The partial oxygen pressure enhances the cell-killing effect, because radiation-induced reactive oxygen species, such as OH radical, initiated DNA breakage (84). Therefore, well-oxygenated areas of a tumor (i.e., the periphery) are killed first, followed by vascularization of the central tumor along with tumor shrinkage. Repetition of this process enhances the efficacy of radiotherapy. The 4Rs reasonably describe the process of tumor shrinkage during radiotherapy and are useful for recognizing tumor and environmental conditions the determine radioresistance or radiosensitivity.

On the other hand, our previous results suggest the possibility that use of the 4Rs might not be appropriate for planning secondary radiotherapy. At the very least, using the same definitions as those used to determine primary radiotherapy might not be suitable for secondary radiotherapy. If the tumor targeted for secondary radiotherapy has acquired radioresistance via EMT, the total dose required to control the tumor should be increased. This suggests that the α and β values of the tumor cells and the dose fractionation used to prevent normal-tissue complications should be changed. Therefore, this suggests that “Repair” and “Repopulation” should be properly adjusted in the planning of secondary radiotherapy. In cases where primary radiotherapy fails to control tumor growth, followed by tumor regrowth within the irradiation field, this suggests that radioresistant cancer cells, such as CSCs, likely exist in the target area. If these tumors are treated with another round of irradiation, “Reoxygenation” might not be suitable for interpreting tumor radiosensitivity, because the CSCs might be in a quiescent state and capable of surviving within the hypoxic area. In addition to the induction of the radioresistant cancer cells, our data showed that repeated photon irradiation *in vivo* promoted acquisition of a more aggressive phenotype in the tumors. These characteristic changes do not fit the classical 4Rs of radiotherapy. Although our results were obtained by experiments using mouse cancer cell lines rather that human specimens, and the results *in vitro* did not match those obtained *in vivo*, they indicated that other hallmarks are required to interpret possible radioresistant or aggressive fractions in target tumors for planning secondary radiotherapy targeting regrown tumors. Given that hypoxic areas are primary niches of CSCs (85), and the tumor vasculature clearly changes after irradiation (86), imaging techniques used to identify hypoxic areas and well-vascularized areas in target tumors will likely be useful for planning secondary radiotherapy. Indeed, drugs targeting hypoxic areas have been developed (87, 88), and tumor blood vessels can be visualized by contrast-enhanced magnetic resonance imaging (89).

## What are the Main Open Questions in the Field?

Although numerous studies show that repeated X-ray irradiation can lead to increased radioresistance in various cancer cells, the primary source of radioresistant cancer cells remains elusive. Therefore, it is imperative to determine whether the selection of inherently radioresistant cells or emergence of radioresistant cells due to *de novo* genetic alterations is the primary cause of the acquisition of radioresistance in order to prevent the appearance of radioresistance and improve patient care. With the recent development of genetic barcoding techniques (90–93), we can now label a large number of cells within a given population. Combined with high-throughput DNA sequencing, genetic barcoding could be used to track and identify the type(s) of cells that can survive and proliferate following repeated irradiation.

Although extensive efforts have been made to investigate the mechanisms of acquisition of radioresistance in cancer cells using *in vitro* models, the radiobiological effects of repeated *in vivo* irradiation remain poorly understood. Tumors are complex ecosystems comprising various cell types, including cancer, stromal, and immune cells. Furthermore, the tumor environment is partly heterogeneous, with hypoxic or nutrient-deprived areas. In this regard, our *in vivo* data suggest that changes in the tumor microenvironment, including angiogenesis, might be critical for the prognosis of mice bearing regrown tumors after repeated irradiation.

In addition to the limited amount of data available concerning the effects of repeated *in vivo* irradiation, the differences between photon and particle irradiation remain largely unknown. Indeed, photon and particle radiation have distinct physical properties, and their resulting biological effects might be very different. For example, particle radiation produces a high density of reactive oxygen species and clustered DNA damage along the particle-beam track (94). Nevertheless, there is, to date, no sensible theory linking the physical characteristics of radiation to their biological effects, including high relative biological effectiveness in cell-survival assays and suppression of metastasis both *in vitro* and *in vivo*.

Answers to these questions would definitely promote the understand of why repeated C-ion irradiation does not appear to induce significant radioresistance in cancer cells, whereas repeated X-ray irradiation leads to significant resistance to both X-ray and C-ion radiation in NR-S1 cells. The identification of potential targets to enhance or elicit radiosensitization could facilitate the development of novel therapeutic strategies for the treatment of radioresistant tumors and recurrent tumors after primary radiotherapy.

## Concluding Remarks

To date, few reports have been published describing the acquisition of radioresistance in repeatedly irradiated tumor cells, particularly after particle irradiation. A series of experiments using models of tumors repeatedly irradiated with either photon or particle radiation show that repeated *in vitro* irradiation with relatively high doses of X-ray radiation can induce significant resistance to both X-ray and C-ion radiation. By contrast, repeated *in vitro* irradiation with relative biological effectiveness doses of C-ion radiation does not contribute to the acquisition of X-ray or C-ion resistance in tumor cells. Somewhat surprisingly, repeated X-ray or C-ion irradiation of *in vivo* regrown tumor models does not increase their radioresistance; however, repeated photon irradiation but not C-ion irradiation increased tumor aggressiveness. Because the evidence was limited to a single tumor cell type, further studies are required to conclusively determine the effects of repeated irradiation on the acquisition of radioresistance in tumors.

## Author Contributions

KS contributed to the design and wrote the manuscript. TS and TI rewrote and made edits. TI conducted the final review.

### Conflict of Interest

The authors declare that the research was conducted in the absence of any commercial or financial relationships that could be construed as a potential conflict of interest.
